# Affecting Effects on Affect: The Impact of Protocol Permutations on Affective Responses to Sprint Interval Exercise; A Systematic Review and Meta-Analysis of Pooled Individual Participant Data

**DOI:** 10.3389/fspor.2022.815555

**Published:** 2022-02-17

**Authors:** Richard S. Metcalfe, Sean Williams, Gwen S. Fernandes, Todd A. Astorino, Matthew J. Stork, Shaun M. Phillips, Ailsa Niven, Niels B. J. Vollaard

**Affiliations:** ^1^Applied Sports Science, Technology, Exercise and Medicine Research Centre (A-STEM), Swansea University, Swansea, United Kingdom; ^2^Department for Health, University of Bath, Bath, United Kingdom; ^3^Population Health Sciences, Bristol Medical School, Bristol, United Kingdom; ^4^Department of Kinesiology, California State University San Marcos, San Marcos, CA, United States; ^5^School of Health and Exercise Sciences, The University of British Columbia, Kelowna, BC, Canada; ^6^Human Performance Science Research Group, University of Edinburgh, Edinburgh, United Kingdom; ^7^Physical Activity for Health Research Centre, University of Edinburgh, Edinburgh, United Kingdom; ^8^Faculty of Health Sciences and Sport, University of Stirling, Stirling, United Kingdom

**Keywords:** sprint interval training, SIT, reduced-exertion high-intensity interval training, REHIT, affective valence, Feeling Scale, meta-analysis, systematic review

## Abstract

Responses to sprint interval exercise (SIE) are hypothesized to be perceived as unpleasant, but SIE protocols are diverse, and moderating effects of various SIE protocol parameters on affective responses are unknown. We performed a systematic search to identify studies (up to 01/05/2021) measuring affective valence using the Feeling Scale during acute SIE in healthy adults. Thirteen studies involving 18 unique trials and 316 unique participant (142 women and 174 men) affective responses to SIE were eligible for inclusion. We received individual participant data for all participants from all studies. All available end-of-sprint affect scores from each trial were combined in a linear mixed model with sprint duration, mode, intensity, recovery duration, familiarization and baseline affect included as covariates. Affective valence decreased significantly and proportionally with each additional sprint repetition, but this effect was modified by sprint duration: affect decreased more during 30 s (0.84 units/sprint; 95% CI: 0.74–0.93) and 15–20 s sprints (1.02 units/sprint; 95% CI: 0.93–1.10) compared with 5–6 s sprints (0.20 units/sprint; 95% CI: 0.18–0.22) (both *p* < 0.0001). Although the difference between 15–20 s and 30 s sprints was also significant (*p* = 0.02), the effect size was trivial (*d* = −0.12). We observed significant but trivial effects of mode, sprint intensity and pre-trial familiarization, whilst there was no significant effect of recovery duration. We conclude that affective valence declines during SIE, but the magnitude of the decrease for an overall SIE session strongly depends on the number and duration of sprints. This information can be applied by researchers to design SIE protocols that are less likely to be perceived as unpleasant in studies of real-world effectiveness.

**Systematic Review Registration:**

Open Science Framework, https://osf.io/sbyn3.

## Introduction

Physical inactivity is a key contributor to the development of multiple chronic diseases, including type 2 diabetes and cardiovascular disease (Booth et al., 2012). However, despite the health benefits of being physically active (Pedersen and Saltin, 2015), it is evident from both self-report and device-driven data capture that a substantial proportion of the general population does not meet the *minimum* recommended thresholds of health-enhancing physical activity (PA) (Troiano et al., 2008; Tucker et al., 2011; Chau et al., 2017; Guthold et al., 2018). It is consistently reported that a lack of time is a key perceived barrier to achieving sufficient PA (Trost et al., 2002; Korkiakangas et al., 2009) and this barrier is in direct conflict with the predominant focus on higher-volume, moderate intensity continuous aerobic activity in PA guidelines (Piercy et al., 2018; UK Chief Medical Officers' Physical Activity Guidelines, 2019). Addressing this perceived barrier of lack of time, has partly driven the proliferation of research in the past 15 years investigating the effects of PA and exercise paradigms that aim to provide health benefits with a reduced exercise and total time commitment, such as high-intensity interval exercise [HIIE; repeated (sub-)maximal efforts] and sprint interval exercise (SIE; repeated “all-out” or supramaximal sprints) (Gibala et al., 2012; Vollaard and Metcalfe, 2017; Gibala and Little, 2019). The accumulated evidence for their efficacy in improving important health biomarkers (Jelleyman et al., 2015; Batacan et al., 2017; Vollaard et al., 2017; Martland et al., 2020) has recently led, for the first time, to reference to very-vigorous-intensity activity and HIIE in UK (UK Chief Medical Officers' Physical Activity Guidelines, 2019) and US (Piercy et al., 2018) PA recommendations.

Nonetheless, performing repeated high intensity efforts or sprints may require high levels of motivation, result in discomfort due to high physical exertion, and cause negative affective responses (Hardcastle et al., 2014; Biddle and Batterham, 2015). Affective responses during exercise are thought to be important because there is some evidence that affective responses during moderate intensity continuous exercise (MICE) may be consequential to future physical activity behavior (Rhodes and Kates, 2015; Brand and Ekkekakis, 2018). Based on studies investigating the effects of exercise intensity on changes in core affective valence during *continuous* exercise, it has been hypothesized that the high exercise intensities prescribed in HIIE and SIE protocols will result in reductions in core affective valence of a greater magnitude than would be observed with MICE (Hardcastle et al., 2014; Biddle and Batterham, 2015). Concomitantly, it may be more likely that people will experience *negative affective valence* (i.e., displeasure) during HIIE/SIE compared to MICE (Hardcastle et al., 2014; Biddle and Batterham, 2015). In partial support of this hypothesis, a recent meta-analysis demonstrated that HIIE and SIE are experienced as significantly less pleasant than MICE (Niven et al., 2020). Consequently, it has been argued that the reductions in core affect during HIIE/SIE could reduce the likelihood of regular engagement in this type of activity (Rhodes and Kates, 2015), although the evidence base is limited, and yet unclear for high intensity activity HIIE and SIE (Stork et al., 2018). Nevertheless, the potential promotion of SIE and/or HIIE as public health interventions is controversial (Hardcastle et al., 2014; Biddle and Batterham, 2015; Rhodes and Kates, 2015). Interestingly, however, there is some evidence that HIIE and SIE are experienced as significantly more enjoyable compared to MICE (Niven et al., 2020).

An important consideration that is often ignored in the debate on the merits of higher-intensity exercise paradigms, is the fact that protocol variations (e.g., number of sprint repetitions, sprint duration, recovery interval duration, training frequency, mode of exercise) make HIIE and SIE interventions highly diverse. Altering any one of these parameters will not only alter the acute whole-body and tissue-specific physiological responses and hence the potential health-related adaptations to training (Metcalfe et al., 2015; Vollaard and Metcalfe, 2017), but also, crucially, could change the affective and perceptual experience during each acute exercise bout. Thus, the affective and perceptual responses to HIIE and SIE, and their impact on exercise enjoyment, motivation, intentions, and adherence, may be more nuanced than is generally described. Treating all HIIE protocols and all SIE protocols, or even all HIIE and SIE protocols combined, as homogeneous entities in meta-analyses will allow conclusions on the *average* protocol but may omit important differences *between* protocols. Indeed, in their recent meta-analysis, Niven et al. (2020) used a definition of HIIE that incorporated both HIIE and SIE, but did not consider the differential effect of each sub-type on affective outcomes. In acknowledging high levels of heterogeneity in the review findings, Niven et al. (2020) recommended that future research should consider the moderating role of protocol variability on affective outcomes. HIIE and SIE are not simply “MICE but harder”; it can be argued that much like resistance exercise, they should be treated as entirely different types of exercise. Similarly, it should be acknowledged that SIE is not just “HIIE but harder”; the exercise intensity during “all-out” supramaximal SIE is several times higher than during (sub-)maximal HIIE, but interval duration can also be several fold shorter. Consequently, acute physiological responses to SIE and HIIE can be different (Wood et al., 2016; Olney et al., 2018). Therefore, we propose that HIIE and SIE should be treated as separate intervention types when examining affective and perceptual responses.

The “classic” early studies on the effects of SIE on health and fitness typically used a protocol involving 4–6 × 30-s all-out sprints with 3–4 min of recovery (Vollaard and Metcalfe, 2017). However, in light of the potential for this specific SIE protocol to cause negative affective responses, more recent research has shifted toward studying the effects of protocols with a lower number of sprint repetitions [2–3; e.g. (Metcalfe et al., 2012; Gillen et al., 2016)] and/or reduced sprint durations [e.g. 5–6 s; e.g. (Adamson et al., 2019, 2020)], on the basis that they may be perceived to be more palatable for previously inactive or unfit individuals (Vollaard and Metcalfe, 2017). There is accumulating evidence that such protocols remain efficacious for improving key biomarkers of cardiovascular and metabolic health in target populations (Metcalfe et al., 2012; Gillen et al., 2016; Vollaard et al., 2017; Adamson et al., 2019, 2020), but an apparent lack of evidence regarding their impact on affective responses. Accordingly, there is considerable merit in establishing how various SIE protocol permutations may impact affective responses during acute SIE and, by extension, which (if any) protocols are most likely to be associated with high levels of adherence. Such findings would help take forward the debate on the public health utility of SIE (and HIIE) interventions. Therefore, in the present study, we performed a systematic review and meta-analysis of all individual participant data from studies that have investigated acute changes in core affective valence during different SIE protocols. Our objective was to determine whether the different SIE protocol parameters modified the affective responses to SIE.

## Methods

The methodology for the current systematic review and meta-analysis was registered on the Open Science Framework (https://osf.io/sbyn3) on the 15^th^ of September 2020 and is reported in accordance with the updated 2020 Preferred Reporting Items for Systematic Reviews and Meta-Analysis (PRISMA) reporting guidelines (Moher et al., 2015). Ethical approval for the review was granted by College of Engineering Research Ethics Committee (reference: 2020-036).

### Literature Search and Study Selection

A literature search of the title and abstract fields for relevant articles written in English was conducted using the databases PubMed, Web of Science, PsychINFO and SPORTDiscus, from inception to 01 May 2021. A modified PICO search was conducted with the following keywords for Exposure: sprint interval, supramaximal, high intensity interval, high intensity intermittent, HIIT, REHIT or interval training. The keywords to define Outcome were: affect^*^, perceptual, pleasure and feeling state. To keep the search parameters broad, we deemed it appropriate not to specify a Comparison group within the search terms. Similarly, the inclusion criteria for Population were broad and defined as healthy human adults 18 years or over and were not specified within the search terms. Search terms for Exposure and Outcome were combined, giving a total of 28 unique search combinations within each database. The electronic database search was supplemented by a manual review of reference lists from relevant articles that were identified within the original database search. All search results were collated into the web-based application Rayyan (Ouzzani et al., 2016), duplicate articles were removed, and then two reviewers (RM, NV) independently screened the resulting articles against pre-defined inclusion and exclusion criteria (outlined below). Articles were first screened by the title and abstract and then full texts were obtained for all articles that appeared to meet the inclusion criteria or where there was any uncertainty following initial title/abstract screening. During the full text review the authors used the label and note functions in Rayyan to highlight eligibility criteria and these were cross-referenced in the event of disagreement in an inclusion/exclusion decision. Any discrepancies in relation to study eligibility were discussed by the two reviewers until a consensus was reached.

### Inclusion and Exclusion Criteria

There are a variety of different tools available to measure core affective valence (Ekkekakis, 2013). Based on previous systematic/scoping reviews of the affective responses to HIIE (Stork et al., 2017; Niven et al., 2020), we anticipated that a range of these tools would have been employed to study the affective responses to SIE. However, these previous reviews (Stork et al., 2017; Niven et al., 2020) also highlighted that the most common tool employed to measure affective valence is Hardy and Rejeski's 11 item Feeling Scale (Hardy and Rejesky, 1989). For example, Niven et al. (2020) reported that The Feeling Scale was employed in 22 out of 33 studies studying the affective responses to HIIE. No other single measure was used by more than 3 studies. Furthermore, several of these scales (e.g. PANAS, POMS) would only be able to be applied post-exercise (Niven et al., 2020), whereas we required a measurement tool that would be able to capture fluctuations in affective valence at multiple time points during SIE. Therefore, as the Feeling Scale is a theoretically appropriate measure to assess the valence dimension of core affect during exercise (Niven et al., 2020), was likely to be the most commonly applied measure, and would ensure consistency of outcome for our pre-planned meta-analysis, we specified our primary outcome as core affective valence measured using the Feeling Scale during acute SIE. In addition, we specified *a priori* that we would only include those studies where affect was measured on immediate completion of more than one of the included sprints (including the final sprint). This decision was taken to capture the most intense portion of the SIE session, as this was likely to be when the lowest affective valence would be reported. We originally intended to perform a qualitative synthesis of studies that measured affective valence with tools other than the Feeling Scale (https://osf.io/sbyn3). However, in order to simplify the interpretation of the effect of SIE protocol parameters on affective valence, we subsequently made the decision (prior to performing final searches) to exclude these and report only on studies that were eligible for our pre-planned meta-analysis.

Considering the aims of this review, it was important to clearly define SIE and how it was differentiated from HIIE. We use the term “interval exercise” as an umbrella term to describe exercise sessions alternating between short periods of relatively intense exercise with periods of relatively lower intensity or resting recovery (Weston et al., 2014), with HIIE and SIE as subcategories of interval exercise. HIIE involves relatively intense but submaximal workloads (based on power at VO_2_max) that elicit between 80 and 100% of maximal heart rate, whilst SIE comprises protocols applying a work intensity >100% of that which elicits maximal oxygen uptake (VO_2_max) during the high-intensity phases, i.e. a “supramaximal” exercise intensity (Weston et al., 2014). However, this definition of SIE is somewhat problematic, because exercise intensities just above those which elicit VO_2_max would hardly meet the dictionary definition of a sprint, that is “*an act of cycling, running etc. at full speed, typically for a limited period of time”*. More importantly, the range of exercise intensities possible within this definition of SIE is substantial; for example, it would not be uncommon for even inactive/unfit individuals to achieve power outputs 2–3.5 times higher than the power at VO_2_max during an “all-out” Wingate style sprint on a cycle ergometer (Ruffino et al., 2017; Metcalfe et al., 2020). For this reason, SIE protocols with such diverse exercise intensities are unlikely to be directly comparable from either a physiological or a psychological perspective. Therefore, although we adopted the currently accepted definition of a work intensity >100% of that which elicits VO_2_max (Weston et al., 2014), the intensity of sprint efforts was extracted from included articles in binary form (i.e., “all out” or “not all out”) and controlled for within the statistical analysis. Protocols that involved workloads less than or equal to 100% of VO_2_max were excluded.

Eligible study designs including randomized cross-over designs with one or more conditions, and intervention studies where affective valence was captured during one or more training sessions during the intervention. Studies that did not measure core affective valence or used a measurement tool other than the Feeling Scale, were excluded, as were studies which did not time their measurement of affect to coincide with the end of sprints, or where affect was only measured in the post-exercise period, as these studies would introduce bias by not capturing the most important (intense) portion of the SIE bout. Studies conducted in healthy human adults (i.e., >18 years of age) were eligible, with studies in children and adolescents, and in patient populations, excluded.

### Data Extraction

Two reviewers (NV, RM) extracted data independently using a standardized spreadsheet and this was subsequently cross-checked for accuracy. The information extracted from the included publications included: study design, study setting, mean participant characteristics, recruitment and retention, details of SIE intervention (i.e., sprint intensity, sprint duration, number of sprint repetitions, recovery duration, recovery intensity, exercise mode, familiarization) and comparators (i.e., intensity, duration, mode etc.), the timings of the affect measurements during SIE, and information to assess the risk of bias. We contacted the corresponding authors via email to request the individual participant data from their study. We requested all raw Feeling Scale data from each time point measured during the SIE condition only. Email correspondence was also used to clarify other data extracted (e.g., the exact timing of affect measurements) where this information was unclear in the published manuscript. Individual participant characteristics were not requested.

### Risk of Bias Assessment

To assess the risk of bias of each included study, we employed the Cochrane Collaboration's risk of bias tool for randomized trials (Higgins et al., 2011). Two reviewers (NV, RM) independently made judgements about risk of bias (using high, low or unclear ratings) for each included study. These were subsequently cross-checked, and any discrepancies were resolved by discussion. The risk of bias judgements were not used to include or exclude studies from any of the planned analyses, but judgements are presented in the results and were considered when drawing overall conclusions from the analyses.

### Data Synthesis

We performed a meta-analysis of the individual participant data to determine the effect of sprint protocol characteristics on affective valence. Our original intention was to convert the affective valence scores during SIE into summary statistics including: (i) the lowest recorded affect score during exercise; and (ii) the change in affective valence (difference between the “pre” and the “lowest” value). However, during the analysis, it became clear that this approach was not optimal to answer the primary research question. Briefly, this is because the design of SIE sessions is such that protocols with longer sprints (e.g., 30 s) typically include a smaller number of sprint repetitions, whereas studies using shorter sprints (5–6 s) typically include a much larger number of sprints. We did not identify examples of protocols involving a small number of shorter sprints, or a high number of longer sprints. Therefore, it was not possible to differentiate the effects of different SIE protocol characteristics in a meta-analysis/meta-regression using a single point summary statistic such as change in affect or lowest reported affect.

**Figure 1:**
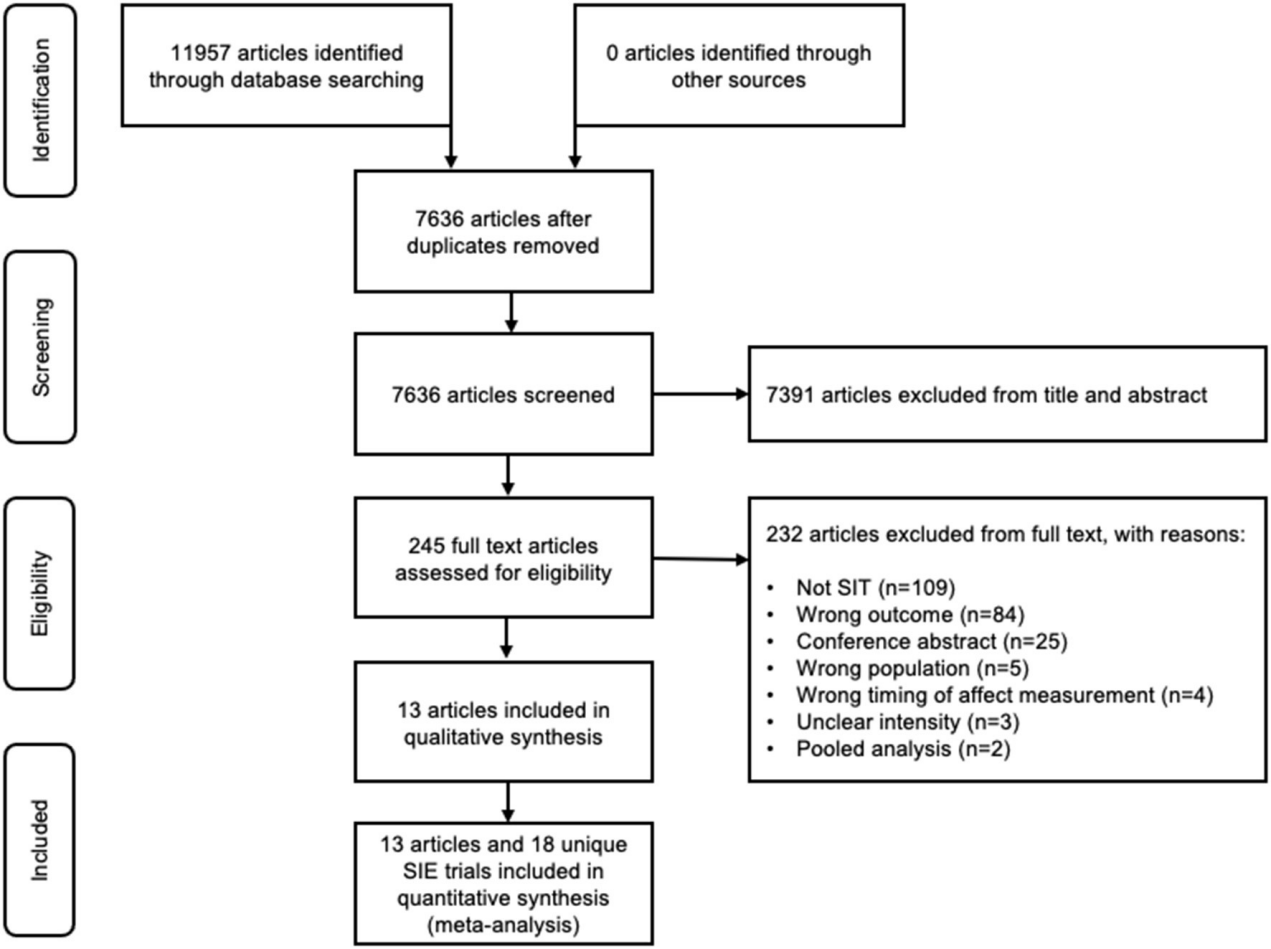
PRISMA flow diagram of the study selection process.

To address this issue, and considering we had retrieved the individual participant level data from each included study, we adopted a one-stage individual participant data (IPD) meta-analysis approach using a general linear mixed model. A visual inspection of the data revealed that the affective responses during SIE were linear for all studies when only the end of sprint timepoints were considered (Figure 1) and, as such, this was deemed to be the most appropriate statistical approach. Affective valence was the dependent variable. The fixed effects in the model were sprint number (numeric linear), sprint duration (5–6 s, 15–20 s, 30 s), and their interaction. Covariates included in the model were baseline affect (numeric linear), recovery duration (numeric linear), mode of exercise (cycling vs. running), sprint intensity (all out vs. not all out), and familiarization (yes vs. no). The random effects were participant ID nested within the study ID. Statistical analysis was performed in R (version 4.0.5, R Foundation for Statistical Computing, Vienna, Austria). Alpha was set at 0.05.

## Results

### Study Selection

**Figure 2:**
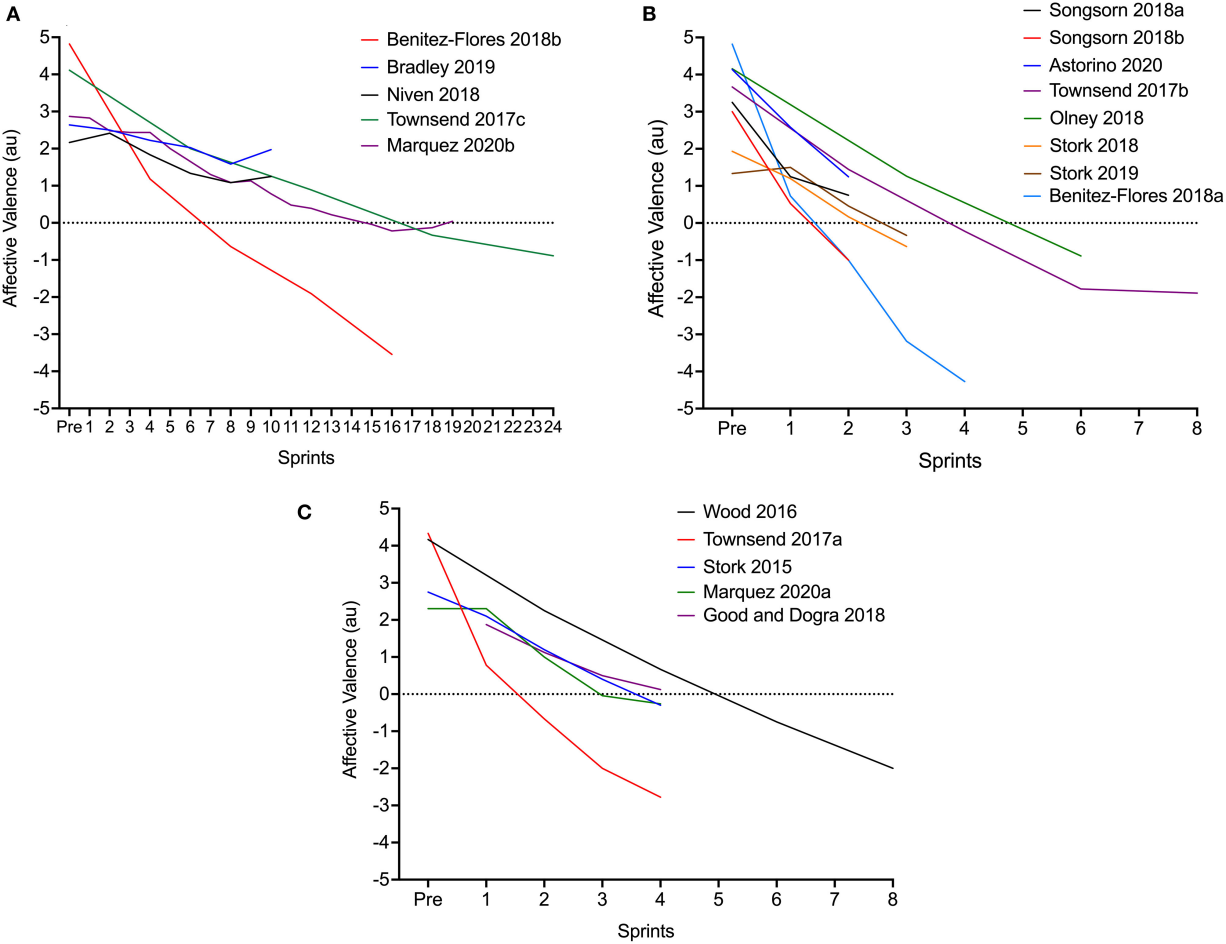
Linear decrease in affective valence during SIE employing short [5–6 s; **(A)**], medium [15–20 s; **(B)**] or long [30-s; **(C)**] sprints.

The database search revealed a total of 11957 titles; after removal of *n* = 4321 duplicates, a total of *n* = 7,636 unique articles were screened. *N* = 7,391 articles were excluded after the initial screening of titles and abstracts, leaving *n* = 245 full text articles for review. Of these full text articles, *n* = 223 were excluded for the following reasons: does not meet definition of SIE (*n* = 109), inappropriate outcome measure (*n* = 84), conference abstract (*n* = 25) or wrong population (*n* = 5). Several other studies (*n* = 9) that aimed to measure the affective response during SIE using The Feeling Scale were excluded because of the following reasons: the timing of the affect measurement was not appropriate for our pre-planned meta-analysis [*n* = 4; (Follador et al., 2018; Sperlich et al., 2018; Haines et al., 2020a,b)], the prescribed exercise intensity of the sprints was unclear [*n* = 3; (Evangelista et al., 2017; Hedlund et al., 2019; Marin et al., 2019)], or because they presented a pooled analysis of published data that was already included [*n* = 2; (Astorino and Vella, 2018; Astorino and Sheard, 2019)]. Finally, one article reported the results from three separate studies conducted in young healthy, inactive individuals (*n* = 2; included) and middle-aged men with type 2 diabetes (*n* = 1; excluded) (Songsorn et al., 2019). In total, *n* = 13 publications incorporating *n* = 18 unique observations were eligible and appropriate for meta-analysis [Figure 2; (Wood et al., 2016; Townsend et al., 2017; Benítez-Flores et al., 2018; Good and Dogra, 2018; Niven et al., 2018; Olney et al., 2018; Bradley et al., 2019; Songsorn et al., 2019; Stork et al., 2019; Astorino et al., 2020; Marques et al., 2020)]. We requested and received full individual participant data for all the eligible studies.

### Study Characteristics

A descriptive overview of the included studies is shown in Table 1. The majority employed acute designs with either a single SIE condition (Bradley et al., 2019; Astorino et al., 2020) or multiple conditions performed in a randomized order (Wood et al., 2016; Townsend et al., 2017; Benítez-Flores et al., 2018; Good and Dogra, 2018; Niven et al., 2018; Olney et al., 2018; Stork et al., 2018; Songsorn et al., 2019; Marques et al., 2020). The studies including multiple conditions either compared SIT to other exercise bouts (Wood et al., 2016; Good and Dogra, 2018; Niven et al., 2018; Olney et al., 2018; Stork et al., 2018; Songsorn et al., 2019), compared various SIE protocol permutations (Townsend et al., 2017; Benítez-Flores et al., 2018; Marques et al., 2020), or investigated the effect of music on the affective response to SIE (Stork et al., 2015, 2019). In studies where the same participants completed different SIE protocol permutations in a cross-over design, each of the conditions were treated as an independent observation. However, in the studies examining the effect of music on affective responses to SIE, only data from the no music condition was included (Stork et al., 2015, 2019). One sub-study involved a 6-week training intervention, with the affective response to the first of the most intense training sessions (week 3; 2 × 20-s all-out cycling sprints) included in the meta-analysis (Songsorn et al., 2019).

**Table 1:** Descriptive overview of studies investigating affective responses to SIE using the feeling scale.

**Reference**	**Study design**	**Sample size and sex**	**Population characteristics (mean** **±SD)**				**Familiarization**	**Sprint interval training protocol**					**Timing of affect measurement**
			**Age (y)**	**BMI**	**Activity status**	**VO_**2**_max**		**Mode**	**Intensity**	**Repetitions**	**Duration**	**Recovery**	
				**(kg/m^**2**^)**		**(ml/kg/min)**				**(n)**	**(s)**	**(s)**
Songsorn et al., 2019	Acute randomized cross over	8 (7 men)	21 ± 1	25 ± 2	Low / Moderately Active (IPAQ)	39 ± 10	Yes	Cycling	All-out	2	20	220	Post every sprint
Songsorn et al., 2019	Training study	19 (10 men)	25 ± 6	24 ± 4	Low / Moderately Active (IPAQ)	34 ± 8	Yes	Cycling	All-out	2	20	220	Post every sprint
Astorino et al., 2020	Acute study with one condition in adults with below (*n =* 43) or above (*n =* 42) average fitness	85 (44 men)	24 ± 7 / 23 ± 4	23 ± 2 / 26 ± 4	Mixed	41 ± 6 / 33 ± 5	No	Cycling	All-out	2	20	180	Post every sprint
Olney et al., 2018	Acute randomized cross over	19 (10 men)	24 ± 3	23 ± 4	Habitually Active	40 ± 6	No	Cycling	140% Wmax	6	20	140	Post sprint 3 and 6
Wood et al., 2016	Acute randomized cross over	12 (8 men)	24 ± 6	24.0	Recreationally Active	41 ± 4	No	Cycling	130% Wmax	8	30	90	Post sprint 2,4,6 and 8
Bradley et al., 2019	Acute study with one condition	36 (12 men)	22 ± 2 / 20 ± 2	24	Mixed	49 ± 4 / 55 ± 2	Yes	Cycling	All-out	10	6	60	Post sprint 2,4,6,8,10
Niven et al., 2018	Acute randomized cross over	12 (12 men)	25 ± 7	24	Active	48 ± 7	No	Cycling	All-out	10	6	60	Post sprint 2,4,6,8, 10
Townsend et al., 2017	Acute randomized cross over	9 (9 men)	23 ± 3	23 ± 4	Recreationally active (<3 sessions/week)	40 ± 6	Yes	Running	All-out	4	30	240	Post every sprint
Townsend et al., 2017	Acute randomized cross over	9 (9 men)	23 ± 3	23 ± 4	Recreationally active (<3 sessions/week)	40 ± 6	Yes	Running	All-out	8	15	120	Post sprint 2,4,5,8
Townsend et al., 2017	Acute randomized cross over	9 (9 men)	23 ± 3	23 ± 4	Recreationally active (<3 sessions/week)	40 ± 6	Yes	Running	All-out	24	5	40	Post sprint 6,12,18,24
Stork et al., 2015	Acute randomized cross over	20 (10 men)	23 ± 4	-	Moderately active (IPAQ)	-	Yes	Cycling	All-out	4	30	240	Post every sprint
Stork et al., 2018	Acute randomized cross over	30 (12 men)	21 ± 4	22 ± 3	Low active (<2 h/week of structured exercise)	31 ± 6	No	Cycling	All-out	3	20	120	Post every sprint
Stork et al., 2019	Acute randomized cross over	24 (12 men)	24 ± 5	23 ± 3	Low active (IPAQ)	39 ± 9	Yes	Cycling	All-out	3	20	120	Post every sprint
Benítez-Flores et al., 2018	Acute randomized cross over	11 (11 men)	26 ± 4	24 ± 1	Highly active (IPAQ)	46 ± 4	Yes	Cycling	All-out	4	20	120	Post every sprint
Benítez-Flores et al., 2018	Acute randomized cross over	11 (11 men)	26 ± 4	24 ± 1	Highly active (IPAQ)	46 ± 4	Yes	Cycling	All-out	16	5	24	Post sprint 4,8,12,16
Marques et al., 2020	Acute randomized cross over	23 (11 men)	25 ± 4	23 ± 3	Low active (IPAQ)	-	No	Running	All-out	4	30	240	Post every sprint
Marques et al., 2020	Acute randomized cross over	23 (11 men)	25 ± 4	23 ± 3	Low active (IPAQ)	-	No	Running	All-out	19	6	40	Post every sprint
Good and Dogra, 2018	Acute randomized cross over	8 (6 men)	22 ± 3	25 ± 5	Active (≥150 minutes of moderate to vigorous intensity exercise per week).	-	No	Cycling	All-out	4	30	270	Post every sprint but no baseline

The included studies involved a total of 316 participants (142 women and 174 men). Fourty three participants (12 women, 11 men) completed more than one of the unique SIE protocols included in the meta-analysis (Townsend et al., 2017; Benítez-Flores et al., 2018; Marques et al., 2020). Except for one study where the inclusion criteria encompassed middle aged adults and the mean BMI was in the overweight range (Astorino et al., 2020), all of the studies were conducted in young, lean adults with a mean age between 20 and 30 years and a mean BMI within the healthy range (Stork et al., 2015, 2018, 2019; Wood et al., 2016; Townsend et al., 2017; Benítez-Flores et al., 2018; Good and Dogra, 2018; Niven et al., 2018; Olney et al., 2018; Bradley et al., 2019; Songsorn et al., 2019; Marques et al., 2020) (Table 1). Physical activity status was more varied: several studies measured physical activity using the International Physical Activity Questionnaire and included participants who scored low (Stork et al., 2019; Marques et al., 2020), low/moderate (Songsorn et al., 2019), moderate (Stork et al., 2015) or highly active (Benítez-Flores et al., 2018). Other studies using more general criteria described participants as low active (<2 h/week of structured exercise) (Stork et al., 2018), recreationally active (<3 sessions/week) (Townsend et al., 2017), active (>150 mins of moderate-vigorous PA per week) (Good and Dogra, 2018), recreationally/habitually active (no criteria) (Wood et al., 2016; Olney et al., 2018), or active but not engaged in a formal training programme (Niven et al., 2018). One study included participants with either a below- or above-average VO_2_max relative to their age (Astorino et al., 2020), whilst one study did not report activity status but mean VO_2_max estimated using a submaximal test was ~50–55 ml/kg/min (Bradley et al., 2019).

A range of different SIE protocols with varying numbers and durations of sprints were employed (Table 1). The majority (*n* = 13) of studies involved cycling (Stork et al., 2015, 2018, 2019; Wood et al., 2016; Benítez-Flores et al., 2018; Good and Dogra, 2018; Niven et al., 2018; Olney et al., 2018; Bradley et al., 2019; Songsorn et al., 2019; Astorino et al., 2020) with the rest (*n* = 5) involving all-out running (Townsend et al., 2017; Marques et al., 2020). The running exercise was performed on a self-propelled treadmill (Townsend et al., 2017) or an outdoor running track (Marques et al., 2020). The cycling was performed all-out on a cycle ergometer: for the majority of studies this was performed against a fixed resistance (5–7.5% body mass) similar to a Wingate sprint (Stork et al., 2015, 2018, 2019; Benítez-Flores et al., 2018; Good and Dogra, 2018; Niven et al., 2018; Bradley et al., 2019; Songsorn et al., 2019; Astorino et al., 2020), however, for two studies the sprints were performed against a resistance of either 130% (Wood et al., 2016) or 140% (Olney et al., 2018) of the power at VO_2_max. A mixture of active (Wood et al., 2016; Benítez-Flores et al., 2018; Good and Dogra, 2018; Olney et al., 2018; Songsorn et al., 2019; Stork et al., 2019; Astorino et al., 2020; Marques et al., 2020) and passive (Stork et al., 2015, 2018; Townsend et al., 2017; Niven et al., 2018; Bradley et al., 2019) recovery was employed. Similarly, several studies provided participants with some familiarization with SIE prior to the main experiment (Stork et al., 2015, 2019; Townsend et al., 2017; Benítez-Flores et al., 2018; Bradley et al., 2019; Songsorn et al., 2019) whilst others did not (Wood et al., 2016; Good and Dogra, 2018; Niven et al., 2018; Olney et al., 2018; Stork et al., 2018; Astorino et al., 2020; Marques et al., 2020).

### Meta-Analysis

Visual inspection of the mean changes in affect during exercise for each observation revealed the decrease in affect with increasing sprint repetitions to be linear (Figure 2). The pooled estimate for the overall effect of sprint number in the linear model when sprint duration was held at the mean value (15.7 s) was −0.54 (95% CI: −0.51 to −0.58) units per additional sprint.

**Figure 3:**
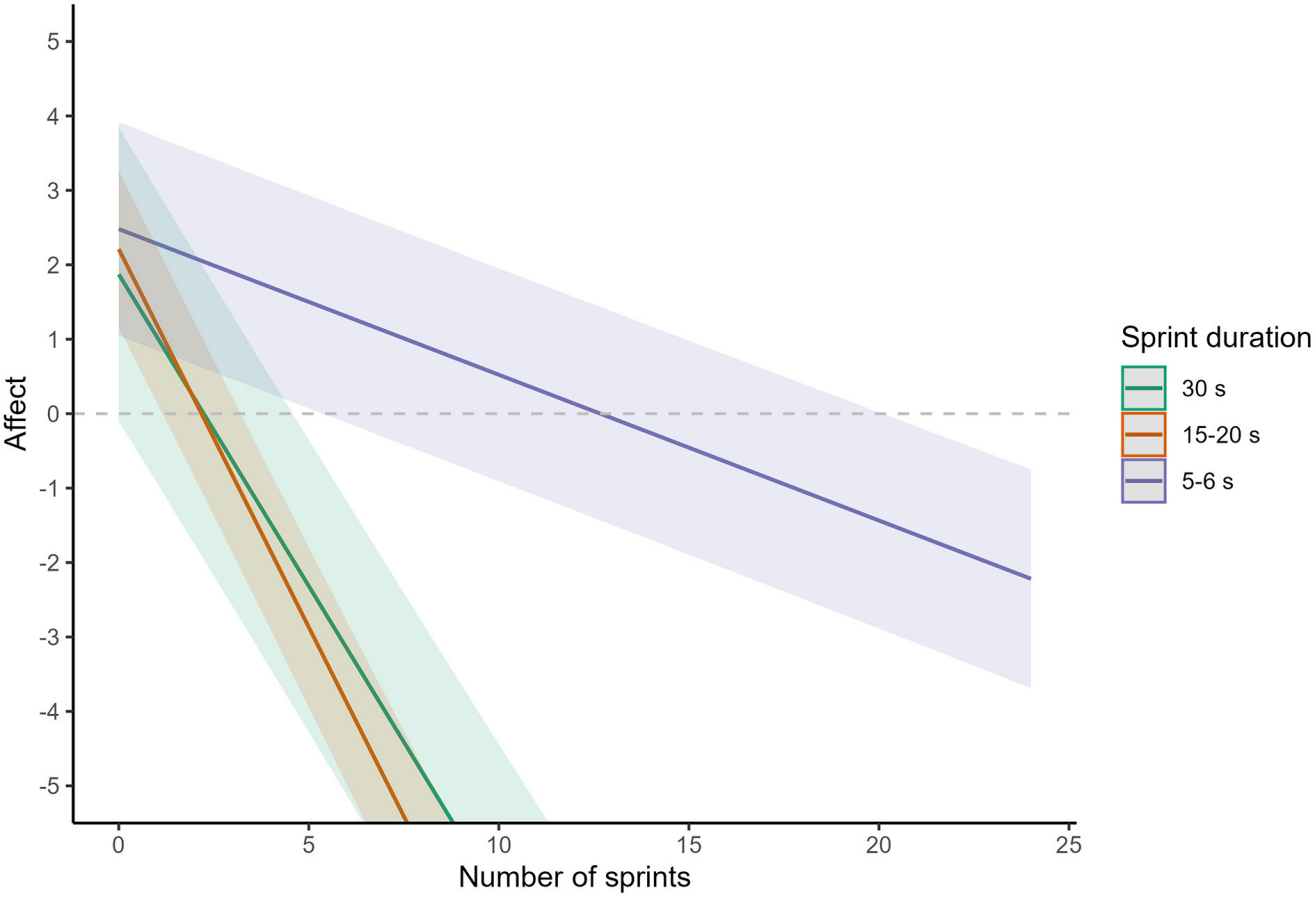
Meta-analysis of the decrease in affective valance during SIE using different sprint durations. The solid line represents the point estimate, whilst the shaded area represents the Bonferroni corrected 95% confidence limits. The purple line and shading are for 5–6 s sprints, the red line and shading are for 15–20 s sprints, and the green line and shading are for 30 s sprints.

The magnitude of the decrease in affect per additional sprint was different when stratified by different sprint durations (Table 2 and Figure 3). For each additional sprint, affect decreased by 0.84 (95% CI: 0.74–0.93) units for 30 s sprints, by 1.02 (95% CI: 0.93–1.10) units for 15–20 s sprints, and by 0.20 (95% CI: 0.18–0.22) units for 5–6 s sprints. Compared to 5–6 s sprints this difference was significant for 30 s (−0.64 units per sprint, 95% CI: −0.52 to −0.76, *p* < 0.0001, *d* = −0.41) and 15–20 s (−0.82 units per sprint, 95% CI: −0.71 to −0.93, *p* < 0.0001, *d* = −0.53). There was also a significantly steeper decline for 15–20 s sprints compared to 30 s sprints (−0.18 units per sprint, 95% CI: −0.33 to −0.02, *p* = 0.02) but the effect size for this difference was trivial (*d* = −0.12).

**Table 2:** Effect of SIE protocol permutations on changes in affective valence during SIE.

	**Estimates**	**95% CIs**	***p*** **-value**
**30 s sprints (reference)**	−0.84	−0.93 – −0.74	7.248e^−73^
*Interactions (estimates per sprint)*			
15–20 s sprints	−0.18	−0.31 – −0.05	0.006
5–6 s sprints	0.64	0.55–0.74	4.257e^−42^
Baseline affect [per 1 unit increase]	−0.09	−0.10 – −0.08	3.564e^−66^
Recovery duration [per 60 sec increase]	0.01	−0.05–0.07	0.467
Mode [running vs. cycling]	0.06	0.02–0.11	0.009
Intensity [not all out vs. all out]	0.18	0.04–0.32	0.009
Familiarization [not familiarized vs. familiarized]	0.06	0.02–0.10	0.002
*Main effects*			
15–20 s sprints	0.34	−1.21–1.89	0.668
5–6 s sprints	0.61	−2.21–3.43	0.673
Baseline affect [per 1 unit increase]	0.40	0.29–0.50	1.476e^−14^
Recovery duration [per 60 sec increase]	0.18	−0.60–0.90	0.656
Mode [running vs. cycling]	0.32	−0.84–1.48	0.587
Intensity [not all out vs. all out]	1.19	−0.88–3.27	0.259
Familiarization [not familiarized vs. familiarized]	0.71	−0.33–1.75	0.183

The slope of the decrease in affect during SIE was also significantly altered by baseline affect, mode, intensity, and familiarization (Table 2). The decrease in affect per sprint was greater when baseline affect was higher (−0.09 units per sprint per 1 unit increase in baseline affect, 95% CI: −0.10 to −0.08, *p* < 0.001), but smaller for running compared to cycling protocols (0.06 units per sprint, 95% CI: 0.02 to 0.11, *p* = 0.009), for “not all out” sprints compared to “all-out” sprints (0.18 units per sprint, 95% CI: 0.04 to 0.32, *p* = 0.009), and in studies that did not familiarize participants with SIE compared with those that did (0.06 units per sprint, 95% CI: 0.02 to 0.10, *p* = 0.002; Table 2). However, the effect sizes for these effects were all trivial (*d*=0.03 to 0.12). The effect of recovery duration was non-significant (Table 2).

All main effects within the model were non-significant, with the exception of baseline affect, where affect was 0.40 units higher throughout SIE for every 1-unit increase in baseline affect (95% CI: 0.29–0.50, *p* < 0.001, Table 2).

### Risk of Bias

**Figure 4:**
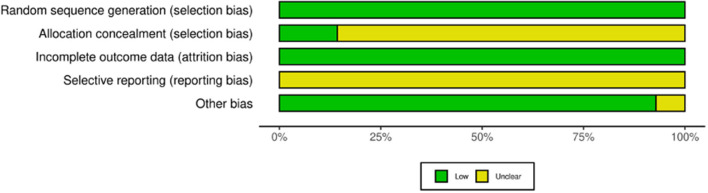
Risk of bias for the included studies.

The risk of bias for all studies is summarized in Figure 4. The risk of selection bias attributable to randomization was low for all studies, either because they were cross-over studies, appropriately randomized, or were studies with a single experimental condition. The risk of selection bias due to allocation concealment was scored as unclear for all studies except those with a single experimental condition. Studies were all scored low risk of bias based on incomplete outcome data, whilst they were all scored unclear for selective reporting because we couldn't find evidence that any of the studies were pre-registered.

## Discussion

The present systematic review and meta-analysis of pooled individual participant data aimed to establish the effect of modifiable protocol parameters on the change in core affective valence during SIE. We show that the primary driver of the change in affect during SIE is the number of sprint repetitions, with affect decreasing progressively and proportionally with each additional sprint repetition that is included in a SIE protocol. However, our findings also demonstrate that sprint duration is a key modifier of this relationship, with a more pronounced decline in affective valence (per sprint) observed for 15–20 and 30 second sprints compared with shorter 5–6 second sprints. Although other protocol parameters also had a significant impact on the decrease in affective valence during SIE, the magnitude of the effects were trivial.

The affective response to acute exercise is thought to be an important predictor of exercise behavior (Ekkekakis and Dafermos, 2012; Rhodes and Kates, 2015; Brand and Ekkekakis, 2018) and the role of SIE as an exercise modality for improving health has been criticized, in part, because SIE is hypothesized to result in negative affective responses (Hardcastle et al., 2014; Biddle and Batterham, 2015). In that context, our findings are important because they demonstrate that not all SIE protocols are the same and the affective response varies depending on SIE protocol design. In turn, our data can be used to determine which SIE protocols will minimize the decline in affective valence during exercise and, therefore, which may be the best SIE protocols to opt for when trying to promote longer term uptake and adherence to SIE in real-world settings.

One of the main aims of SIE protocols is generally to provide a time-efficient alternative to both MICE and HIIE for improving health (Vollaard and Metcalfe, 2017). As lowering the number of sprint repetitions is one of the most effective ways to reduce overall training time-commitment, the number of sprint repetitions is a key consideration in SIE protocol design (Vollaard and Metcalfe, 2017). The present data demonstrate that as well as reducing time-commitment, an additional benefit of reducing the number of sprint repetitions will be a smaller in-task decrease in core affective valence. Based on the mean baseline affect of 3.2 units for all individuals included in this meta-analysis, two longer (15–30 s) sprints or up to ten shorter (5–6 s) sprints will reduce affective valence to ~1 unit on the Feeling Scale. Thus, although SIE will lead to reduced valence, our analysis shows that *negative* valence could be avoided with SIE protocols with a total time commitment of up to ~10 min per session (Niven et al., 2018; Bradley et al., 2019; Songsorn et al., 2019; Astorino et al., 2020). This is important, as there is an accumulating body of evidence demonstrating that these SIE protocols are still associated with meaningful improvements in cardiorespiratory fitness as well as other markers of cardiometabolic health (Metcalfe et al., 2012; Vollaard et al., 2017; Adamson et al., 2019, 2020). Overall, this meta-analysis provides further evidence that SIE protocols should employ a minimal number of sprint repetitions, as this (1) improves time-efficiency, (2) attenuates the decrease in affective valence, and (3) does not appear to impact health related adaptations to training. Future studies should establish the most time-efficient protocols that are still associated with meaningful health benefits.

In addition to the number of sprint repetitions, our analysis demonstrates that sprint duration is another important factor when considering changes in affect with SIE. For a given number of sprint repetitions, affective valence is always more positive following 5–6-s sprints compared to 15–30-s sprints. This difference increases in magnitude when the number of sprint repetitions increases. However, when considering the practical implications of this finding, it is important to note that there is evidence that reducing sprint duration attenuates the improvements in cardiorespiratory fitness when the number of sprint repetitions is low (Nalçakan et al., 2018). As a result, a greater number of repetitions may be required when applying sprints of shorter duration and it may be more appropriate to consider the effect of sprint duration on affective valence when matched for total sprint volume. Our meta-analysis suggest that 4–5 shorter (5–6 s) sprints result in a similar decrease in affective valence compared with 1 longer (20–30 s) sprint (~1 unit on the Feeling Scale). Based on these estimates, overall reductions in affective valence are likely to be broadly similar between shorter and longer sprints if total sprint volume is matched. However, it is worth noting that some studies that have made direct within-subject comparisons of different sprint durations when matched for sprint volume [two of which could not be included in the current meta-analysis due to the timing of affect measurements (Haines et al., 2020a,b)] have found more positive affective valence with shorter compared with longer sprints (Townsend et al., 2017; Haines et al., 2020a,b). In addition, there is some evidence that participants report a preference for and greater intentions to engage in SIE protocols involving shorter compared with longer sprint durations (Townsend et al., 2017; Metcalfe et al., 2020). Together, the currently available evidence suggests that decreasing the sprint duration in an SIE protocol may result in more positive affective responses, but care should be taken to avoid potential reductions in the efficacy to improve key health biomarkers.

We also found that the slope of the decrease in affect during SIE was less steep for running compared with cycling protocols, and when sprint intensity was not all-out compared to all-out. In both instances, this is likely to be explained by lower power output during the sprints. However, the magnitude of the effect of these parameters was small and therefore their real-world practical relevance is unclear. In addition, the number of studies examining these protocol variations was low, meaning the analysis may have been underpowered, and therefore some caution is warranted when interpreting these results. Interestingly, we also found no effect of recovery duration on the affective response to SIE when controlling for sprint duration. This contrasts with what might be expected; intuitively, longer recovery durations would be expected to attenuate the decrease in affective valence, whilst shorter recovery durations would be expected to result in a more pronounced decrease. The lack of impact of recovery duration in our analysis may be explained by the lack of variation in recovery durations between studies that had used similar sprint durations, e.g., studies using 30-s sprints tended to use recovery durations lasting ~240 s (Table 1). Overall, this systematic review has revealed an opportunity for future studies to examine the effect of different exercise modes, sprint intensities and recovery durations on the affective response to SIE. Considering the clear, large effect of sprint number and sprint duration on the affective response, we would particularly encourage researchers to study the effect of these protocol parameters within SIE protocols utilizing a small number of longer sprints, or a larger number of shorter sprints (i.e., SIE protocols where affective response are likely to remain positive on average).

It is important to point out that all but one of the studies included in this review were conducted in young (mean age between 20 and 30 years) populations, free from chronic disease, and with a healthy weight status (mean BMI <25 kg/m^2^). Furthermore, although a number of studies were conducted with participants not meeting current exercise guidelines (i.e., insufficiently active), several were conducted in active populations. This can be considered a strength because it means that differences in the affective response between studies can be ascribed to the SIE protocols rather than differences between the study populations. However, it is also a limitation, because it means that our findings cannot necessarily be translated beyond these populations. Therefore, an important finding of this systematic review is the need for future work to illustrate the effect of different participant characteristics on the affective responses to SIE. Whilst we would anticipate that the overall effect of the different SIE protocol permutations identified in this meta-analysis are likely to be similar across different populations, it can be speculated that the magnitude of the decrease in affective valence (per sprint) may be greater in less fit, overweight/obese populations or in those living with chronic disease associated poorer exercise tolerance (e.g. type 2 diabetes). However, this is not a foregone conclusion as previous research has reported mixed findings of participant characteristics on the affective response to SIE (Saanijoki et al., 2018; Astorino et al., 2020). Specifically, whilst Saanijoki et al. (2018) reported more negative affective responses to SIE (4 × 30 s all-out sprints) in middle aged people with insulin resistance compared to healthy middled aged individuals, Astorino et al. (2020) found no difference in the affective response to SIE (2 × 20 s all-out sprints) between people with below compared to above average levels of cardiorespiratory fitness. Thus, there is a need for future studies to assess the effect of participant characteristics on changes in affective valence with SIT. This would help establish the suitability of specific SIT protocols for different populations.

## Perspectives and Conclusions

The majority of SIE research to date has focused on studying the efficacy of SIE to improve health and fitness. This has led to a large body of evidence supporting that, within the wide variety of studied SIE protocols, most appear to have at least some positive effects on key health biomarkers (Vollaard and Metcalfe, 2017; Vollaard et al., 2017). However, in order to significantly impact public health, a meaningful proportion of individuals need to be willing to adopt and adhere to SIE in real-world environments. As the affective response to exercise is thought to be a potentially important predictor of exercise adherence (Ekkekakis and Dafermos, 2012; Brand and Ekkekakis, 2018), the present meta-analysis takes an important step forward in assessing which SIE interventions are most likely to be associated with positive valence, and therefore may lead to higher levels of adherence.

Although our findings demonstrate that affective valence does decrease during SIE, the extent to which it decreases and whether a person is likely to experience *negative* valence (i.e. displeasure) varies between different SIE protocols, and is particularly dependent on the interaction between the number and duration of sprint efforts. Our data indicate that positive valence can be maintained during SIE by using up to 2 repetitions of longer sprint durations [15–30 s; termed “reduced-exertion high-intensity interval training” (REHIT) (Metcalfe et al., 2012, 2015, 2016; Ruffino et al., 2017; Nalçakan et al., 2018; Thomas et al., 2020)] or by using multiple sprints of shorter duration [5–6 s; (Niven et al., 2018; Bradley et al., 2019)]. Each of these SIE protocol variants has evidence for their efficacy at improving key health markers (Metcalfe et al., 2012, 2016; Adamson et al., 2014, 2019, 2020; Ruffino et al., 2017; Vollaard et al., 2017; Nalçakan et al., 2018; Thomas et al., 2020), whilst recent pilot work has shown that REHIT may be feasible and effective in a ‘real-world' workplace setting in the short term (Metcalfe et al., 2020). There remains a need for future research to assess the longer-term effectiveness of SIE (and HIIE) for improving health. However, our current data add further weight to the suggestion that these studies should focus on SIE protocols that utilize minimal sprint durations and repetitions (Vollaard and Metcalfe, 2017), as this appears to be one of the best strategies to identify acceptable, time-efficient exercise interventions for improving health.

## Data Availability Statement

The original contributions presented in the study are included in the article/supplementary materials, further inquiries can be directed to the corresponding author/s.

## Author Contributions

The study was conceived by NV and RM. NV, RM, SW, and GF contributed to the design of the study. RM and NV drafted the manuscript for publication, while all authors contributed to critically reviewing the manuscript. All authors contributed to data acquisition and interpretation of data and gave final approval of the manuscript for publication.

## Conflict of Interest

The authors declare that the research was conducted in the absence of any commercial or financial relationships that could be construed as a potential conflict of interest.

## Publisher's Note

All claims expressed in this article are solely those of the authors and do not necessarily represent those of their affiliated organizations, or those of the publisher, the editors and the reviewers. Any product that may be evaluated in this article, or claim that may be made by its manufacturer, is not guaranteed or endorsed by the publisher.

## References

[B1] Adamson S. Kavaliauskas M. Lorimer R. Babraj J. (2020). The impact of sprint interval training frequency on blood glucose control and physical function of older adults. Int. J. Environ. Res. Public. Health. 17, 454. 10.3390/ijerph17020454 31936725 PMC7013863

[B2] Adamson S. Kavaliauskas M. Yamagishi T. Phillips S. Lorimer R. Babraj J. (2019). Extremely short duration sprint interval training improves vascular health in older adults. Sport Sci. Health 15, 123–131. 10.1007/s11332-018-0498-2

[B3] Adamson S. Lorimer R. Cobley J. N. Lloyd R. Babraj J. (2014). High intensity training improves health and physical function in middle aged adults. Biology. 3, 333–344. 10.3390/biology3020333 24833513 PMC4085611

[B4] Astorino T. A. Clausen R. Marroquin J. Arthur B. Stiles K. (2020). Similar perceptual responses to reduced exertion high intensity interval training (REHIT) in adults differing in cardiorespiratory fitness. Physiol. Behav. 11, 2687. 10.1016/j.physbeh.2019.112687 31622613

[B5] Astorino T. A. Sheard A. C. (2019). Does sex mediate the affective response to high intensity interval exercise? Physiol. Behav. 204, 27–32. 10.1016/j.physbeh.2019.02.005 30738970

[B6] Astorino T. A. Vella C. A. (2018). Predictors of change in affect in response to high intensity interval exercise (HIIE) and sprint interval exercise (SIE). Physiol. Behav. 196, 211–217. 10.1016/j.physbeh.2018.08.017 30170171

[B7] Batacan R. B. Duncan M. J. Dalbo V. J. Tucker P. S. Fenning A. S. (2017). Effects of high-intensity interval training on cardiometabolic health: a systematic review and meta-analysis of intervention studies. Br. J. Sports Med. 51, 494–503. 10.1136/bjsports-2015-095841 27797726

[B8] Benítez-Flores S. de Sousa A. F. M. da Cunha Totó E. C. Santos Rosa T. Del Rosso S. Foster C. . (2018). Shorter sprints elicit greater cardiorespiratory and mechanical responses with less fatigue during time-matched sprint interval training (SIT) sessions. Kinesiology. 50, 137–148. 10.26582/k.50.2.13

[B9] Biddle S. J. Batterham A. M. (2015). High-intensity interval exercise training for public health: a big HIT or shall we HIT it on the head? Int J Behav Nutr Phys Act 12, 95. 10.1186/s12966-015-0254-9 26187579 PMC4506613

[B10] Booth F. W. Roberts C. K. Laye M. J. (2012). Lack of exercise is a major cause of chronic diseases. Compr Physiol. 2, 1143–1211. 10.1002/cphy.c110025 23798298 PMC4241367

[B11] Bradley C. Niven A. Phillips S. M. (2019). Self-reported tolerance of the intensity of exercise influences affective responses to and intentions to engage with high-intensity interval exercise. J. Sports Sci. 37, 1472–1480. 10.1080/02640414.2019.1570590 30694110

[B12] Brand R. Ekkekakis P. (2018). Affective-reflective theory of physical inactivity and exercise: foundations and preliminary evidence. Ger. J. Exerc. Sport Res. 48, 48–58. 10.1007/s12662-017-0477-9

[B13] Chau J. Chey T. Burks-Young S. Engelen L. Bauman A. (2017). Trends in prevalence of leisure time physical activity and inactivity: results from Australian National Health Surveys 1989 to 2011. Aust. N. Z. J. Public Health 41, 617–624. 10.1111/1753-6405.12699 28749561

[B14] Ekkekakis P.. (2013). The Measurement of Affect, Mood, and Emotion: A Guide for Health-Behavioral Research. Cambridge University Press. 10.1017/CBO9780511820724

[B15] Ekkekakis P. Dafermos M. (2012). Exercise Is a Many-Splendored Thing, but for Some It Does Not Feel So Splendid: Staging a Resurgence of Hedonistic Ideas in the Quest to Understand Exercise Behavior. Oxf. Handb. Exerc. Psychol. 10.1093/oxfordhb/9780195394313.013.00166446231

[B16] Evangelista A. L. Rica R. L. Fernandes A. Miranda J. M. Q. Scala C. V. L. Lopes C. R. . (2017). Effects of high-intensity calisthenic training on mood and affective responses. J. Exerc. Physiol. 20, 15–23.

[B17] Follador L. Alves R. C. Ferreira S. D. S. Buzzachera C. F. Andrade V. F. D. S. Garcia E. D. S. . (2018). Physiological, perceptual, and affective responses to six high-intensity interval training protocols. Percept. Mot. Skills 125, 329–350. 10.1177/0031512518754584 29368530

[B18] Gibala M. J. Little J. P. (2019). Physiological basis of brief vigorous exercise to improve health. J. Physiol. 598, 61–69. 10.1113/JP276849 31691289

[B19] Gibala M. J. Little J. P. Macdonald M. J. Hawley J. A. (2012). Physiological adaptations to low-volume, high-intensity interval training in health and disease. J Physiol. 590, 1077–1084. 10.1113/jphysiol.2011.224725 22289907 PMC3381816

[B20] Gillen J. B. Martin B. J. MacInnis M. J. Skelly L. E. Tarnopolsky M. A. Gibala M. J. (2016). Twelve weeks of sprint interval training improves indices of cardiometabolic health similar to traditional endurance training despite a five-fold lower exercise volume and time commitment. PLoS ONE. 11, e0154075. 10.1371/journal.pone.0154075 27115137 PMC4846072

[B21] Good J. Dogra S. (2018). Subjective responses to sprint interval exercise in adults with and without Exercise-induced bronchoconstriction. J. Asthma. 55, 1059–1067. 10.1080/02770903.2017.1391282 29023174

[B22] Guthold R. Stevens G. A. Riley L. M. Bull F. C. (2018). Worldwide trends in insufficient physical activity from 2001 to 2016: a pooled analysis of 358 population-based surveys with 1·9 million participants. Lancet Glob. Health 6, e1077–e1086. 10.1016/S2214-109X(18)30357-7 30193830

[B23] Haines M. Broom D. Gillibrand W. Stephenson J. (2020a). Effects of three low-volume, high-intensity exercise conditions on affective valence. J. Sports Sci. 38, 121–129. 10.1080/02640414.2019.1684779 31661663

[B24] Haines M. Broom D. Stephenson J. Gillibrand W. (2020b). Influence of sprint duration during minimal volume exercise on aerobic capacity and affect. Int. J. Sports Med. a-1255–3161. 10.1055/a-1255-3161 33022736

[B25] Hardcastle S. J. Ray H. Beale L. Hagger M. S. (2014). Why sprint interval training is inappropriate for a largely sedentary population. Front. Psychol 5, 1505. 10.3389/fpsyg.2014.01505 25566166 PMC4274872

[B26] Hardy C. J. Rejesky J. W. (1989). Not what, but how one feels: the measurement of affect during exercise. J. Sport Exerc. Psychol. 11, 304–317. 10.1123/jsep.11.3.304

[B27] Hedlund M. Lindelöf N. Johansson B. Boraxbekk C.-J. Rosendahl E. (2019). Development and feasibility of a regulated, supramaximal high-intensity training program adapted for older individuals. Front. Physiol. 10, 590. 10.3389/fphys.2019.00590 31164835 PMC6536694

[B28] Higgins J. P. T. Altman D. G. Gøtzsche P. C. Jüni P. Moher D. Oxman A. D. . (2011). The Cochrane Collaboration's tool for assessing risk of bias in randomised trials. BMJ. 343, d5928. 10.1136/bmj.d5928 22008217 PMC3196245

[B29] Jelleyman C. Yates T. O'Donovan G. Gray L. J. King J. A. Khunti K. . (2015). The effects of high-intensity interval training on glucose regulation and insulin resistance: a meta-analysis: the effects of HIIT on metabolic health. Obes. Rev. 16, 942–961. 10.1111/obr.12317 26481101

[B30] Korkiakangas E. E. Alahuhta M. A. Laitinen J. H. (2009). Barriers to regular exercise among adults at high risk or diagnosed with type 2 diabetes: a systematic review. Health Promot. Int. 24, 416–427. 10.1093/heapro/dap031 19793763

[B31] Marin D. P. Astorino T. A. Martinatto F. Ragazzini F. T. Bispo R. E. Foschini D. . (2019). Comparison of perceptual responses between different upper-body sprint interval exercise protocols. Physiol. Behav. 210, 112626. 10.1016/j.physbeh.2019.112626 31344392

[B32] Marques M. Alves E. Henrique N. Franchini E. (2020). Positive affective and enjoyment responses to four high-intensity interval exercise protocols. Percept. Mot. Skills. 127, 742–765. 10.1177/0031512520918748 32323607

[B33] Martland R. Mondelli V. Gaughran F. Stubbs B. (2020). Can high intensity interval training improve health outcomes among people with mental illness? A systematic review and preliminary meta-analysis of intervention studies across a range of mental illnesses. J. Affect. Disord. 263, 629–660. 10.1016/j.jad.2019.11.039 31780128

[B34] Metcalfe R. S. Atef H. Mackintosh K. McNarry M. Ryde G. Hill D. M. . (2020). Time-efficient and computer-guided sprint interval exercise training for improving health in the workplace: a randomised mixed-methods feasibility study in office-based employees. BMC Public Health 20, 313. 10.1186/s12889-020-8444-z 32164631 PMC7068982

[B35] Metcalfe R. S. Babraj J. A. Fawkner S. G. Vollaard N. B. (2012). Towards the minimal amount of exercise for improving metabolic health: beneficial effects of reduced-exertion high-intensity interval training. Eur. J. Appl. Physiol. 112, 2767–2775. 10.1007/s00421-011-2254-z 22124524

[B36] Metcalfe R. S. Koumanov F. Ruffino J. S. Stokes K. A. Holman G. D. Thompson D. . (2015). Physiological and molecular responses to an acute bout of reduced-exertion high-intensity interval training (REHIT). Eur. J. Appl. Physiol. 115, 2321–2334. 10.1007/s00421-015-3217-6 26156806

[B37] Metcalfe R. S. Tardif N. Thompson D. Vollaard N. B. (2016). Changes in aerobic capacity and glycaemic control in response to reduced-exertion high-intensity interval training (REHIT) are not different between sedentary men and women. Appl. Physiol. Nutr. Metab. 41, 1117–1123. 10.1139/apnm-2016-0253 27753506

[B38] Moher D. Shamseer L. Clarke M. Ghersi D. Liberati A. Petticrew M. . (2015). Preferred reporting items for systematic review and meta-analysis protocols (PRISMA-P) 2015 statement. Syst. Rev. 4, 1. 10.1186/2046-4053-4-1 25554246 PMC4320440

[B39] Nalçakan G. R. Songsorn P. Fitzpatrick B. L. Yüzbasioglu Y. Brick N. E. Metcalfe R. S. . (2018). Decreasing sprint duration from 20 to 10 s during reduced-exertion high-intensity interval training (REHIT) attenuates the increase in maximal aerobic capacity but has no effect on affective and perceptual responses. Appl. Physiol. Nutr. Metab. 43, 338–344. 10.1139/apnm-2017-0597 29172029

[B40] Niven A. Laird Y. Saunders D. H. Phillips S. M. (2020). A systematic review and meta-analysis of affective responses to acute high intensity interval exercise compared with continuous moderate- and high-Intensity exercise. Health Psychol. Rev. 15, 540–573. 10.1080/17437199.2020.1728564 32067574

[B41] Niven A. Thow J. Holroyd J. Turner A. P. Phillips S. M. (2018). Comparison of affective responses during and after low volume high-intensity interval exercise, continuous moderate- and continuous high-intensity exercise in active, untrained, healthy males. J. Sports Sci. 36, 1993–2001. 10.1080/02640414.2018.1430984 29376774

[B42] Olney N. Wertz T. LaPorta Z. Mora A. Serbas J. Astorino T. A. (2018). Comparison of acute physiological and psychological responses between moderate intensity continuous exercise and three regimes of high intensity training. J. Strength Cond. Res. 32, 2130–2138. 10.1519/JSC.0000000000002154 28737586

[B43] Ouzzani M. Hammady H. Fedorowicz Z. Elmagarmid A. (2016). Rayyan—a web and mobile app for systematic reviews. Syst. Rev. 5:210. 10.1186/s13643-016-0384-4 27919275 PMC5139140

[B44] Pedersen B. K. Saltin B. (2015). Exercise as medicine - evidence for prescribing exercise as therapy in 26 different chronic diseases. Scand. J. Med. Sci. Sports 25 Suppl 3, 1–72. 10.1111/sms.12581 26606383

[B45] Piercy K. L. Troiano R. P. Ballard R. M. Carlson S. A. Fulton J. E. Galuska D. A. . (2018). The physical activity guidelines for americans. JAMA. 320, 2020–2028. 10.1001/jama.2018.14854 30418471 PMC9582631

[B46] Rhodes R. E. Kates A. (2015). Can the affective response to exercise predict future motives and physical activity behavior? A systematic review of published evidence. Ann. Behav. Med. 49, 715–731. 10.1007/s12160-015-9704-5 25921307

[B47] Ruffino J. S. Songsorn P. Haggett M. Edmonds D. Robinson A. M. Thompson D. . (2017). A comparison of the health benefits of reduced-exertion high-intensity interval training (REHIT) and moderate-intensity walking in type 2 diabetes patients. Appl. Physiol. Nutr. Metab. 42, 202–208. 10.1139/apnm-2016-0497 28121184

[B48] Saanijoki T. Nummenmaa L. Koivumäki M. Löyttyniemi E. Kalliokoski K. K. Hannukainen J. C. (2018). Affective Adaptation to Repeated SIT and MICT Protocols in Insulin-Resistant Subjects. Med. Sci. Sports Exerc. 50, 18–27. 10.1249/MSS.0000000000001415 28857909

[B49] Songsorn P. Brick N. Fitzpatrick B. Fitzpatrick S. McDermott G. McClean C. . (2019). Affective and perceptual responses during reduced-exertion high-intensity interval training (REHIT). Int. J. Sport Exerc. Psychol. 18, 717–732. 10.1080/1612197X.2019.159321729172029

[B50] Sperlich B. De Clerck I. Zinner C. Holmberg H.-C. Wallmann-Sperlich B. (2018). Prolonged sitting interrupted by 6-min of high-intensity exercise: circulatory, metabolic, hormonal, thermal, cognitive, and perceptual responses. Front. Physiol. 9, 1279. 10.3389/fphys.2018.01279 30386249 PMC6198043

[B51] Stork M. J. Banfield L. E. Gibala M. J. Martin Ginis K. A. (2017). A scoping review of the psychological responses to interval exercise: is interval exercise a viable alternative to traditional exercise? Health Psychol. Rev. 11, 324–344. 10.1080/17437199.2017.1326011 28460601

[B52] Stork M. J. Gibala M. J. Martin Ginis K. A. (2018). Psychological and behavioral responses to interval and continuous exercise. Med. Sci. Sports Exerc. 50, 2110–2121. 10.1249/MSS.0000000000001671 29771824

[B53] Stork M. J. Karageorghis C. I. Martin Ginis K. A. (2019). Let's Go: psychological, psychophysical, and physiological effects of music during sprint interval exercise. Psychol. Sport Exerc. 45, 101547. 10.1016/j.psychsport.2019.101547

[B54] Stork M. J. Kwan M. Y. W. Gibala M. J. Martin Ginis K. A. (2015). Music enhances performance and perceived enjoyment of sprint interval exercise. Med. Sci. Sports Exerc. 47, 1052–1060. 10.1249/MSS.0000000000000494 25202850

[B55] Thomas G. Songsorn P. Gorman A. Brackenridge B. Cullen T. Fitzpatrick B. . (2020). Reducing training frequency from 3 or 4 sessions/week to 2 sessions/week does not attenuate improvements in maximal aerobic capacity with reduced-exertion high-intensity interval training (REHIT). Appl. Physiol. Nutr. Metab. 45, 683–685. 10.1139/apnm-2019-0750 32078337

[B56] Townsend L. K. Islam H. Dunn E. Eys M. Robertson-Wilson J. Hazell T. J. (2017). Modified sprint interval training protocols. Part II. Psychological responses. Appl. Physiol. Nutr. Metab. 42, 347–353. 10.1139/apnm-2016-0479 28177741

[B57] Troiano R. P. Berrigan D. Dodd K. W. Mâsse L. C. Tilert T. McDowell M. (2008). Physical activity in the United States measured by accelerometer. Med. Sci. Sports Exerc. 40, 181–188. 10.1249/mss.0b013e31815a51b3 18091006

[B58] Trost S. G. Owen N. Bauman A. E. Sallis J. F. Brown W. (2002). Correlates of adults' participation in physical activity: review and update. Med. Sci. Sports Exerc. 34, 1996–2001. 10.1097/00005768-200212000-00020 12471307

[B59] Tucker J. M. Welk G. J. Beyler N. K. (2011). Physical activity in U.S.: adults compliance with the Physical Activity Guidelines for Americans. Am. J. Prev. Med. 40, 454–61. 10.1016/j.amepre.2010.12.016 21406280

[B60] UK Chief Medical Officers' Physical Activity Guidelines (2019). Available online at: https://www.gov.uk/government/publications/physical-activity-guidelines-uk-chief-medical-officers-report. (accessed November 12, 2021).

[B61] Vollaard N. B. Metcalfe R. S. (2017). Research into the health benefits of sprint interval training should focus on protocols with fewer and shorter sprints. Sports Med 47, 2443–2451. 10.1007/s40279-017-0727-x 28391489 PMC5684281

[B62] Vollaard N. B. J. Metcalfe R. S. Williams S. (2017). Effect of number of sprints in an SIT session on change in V?O2max: a meta-analysis. Med. Sci. Sports Exerc. 49, 1147–1156. 10.1249/MSS.0000000000001204 28079707

[B63] Weston K. S. Wisløff U. Coombes J. S. (2014). High-intensity interval training in patients with lifestyle-induced cardiometabolic disease: a systematic review and meta-analysis. Br. J. Sports Med. 48, 1227–1234. 10.1136/bjsports-2013-092576 24144531

[B64] Wood K. M. Olive B. LaValle K. Thompson H. Greer K. Astorino T. A. (2016). Dissimilar physiological and perceptual responses between sprint interval training and high-intensity interval training. J. Strength Cond. Res. 30, 244–250. 10.1519/JSC.0000000000001042 26691413

